# Characterization of Specific RAPD Markers of Virulence in *Trichomonas vaginalis* Isolates

**Published:** 2015

**Authors:** Jorge FRAGA, Lázara ROJAS, Idalia SARIEGO, Aymé FERNÁNDEZ-CALIENES

**Affiliations:** *Dept. of Parasitology, Institute of Tropical Medicine Pedro Kourí, Havana, Cuba *

**Keywords:** *Trichomonas vaginalis*, RAPD, Virulence marker, *Trichomonas vaginalis virus*

## Abstract

***Background:*** As for human trichomoniasis the host-parasite relationship is very complex, and the broad ranges of clinical symptoms are unlikely be attributable to a single pathogenic mechanism. Specific Random Amplified Polymorphic DNA (RAPD) markers of 490 bp, 720 bp and 460 bp using the primers Tv-5, OPA-6 and OPA-11, respectively, were reported. This was the first description of possible genetic virulence markers of the infection by *T. vaginalis*. The aim of this study was to characterize the specific RAPD markers in order to elucidate their importance on virulence of this illness.

***Methods:*** The selected specific RAPD fragments were cloned and sequenced. The obtained sequences were compared by the BLAST algorithm.

***Results:*** The nucleotide sequence of the Tv-5_490 _RAPD marker exhibited significant similarity to *T. vaginalis* hypothetical G3 leucine rich repeat (LRR) family protein (e-value: 6e-14) and *Giardia lamblia* leucine rich repeat protein 1 virus receptor protein (e-value: 6e-14 and 2e-12) ; however, the OPA-6_720_ and OPA-11_460_ showed no significant similarity with any coding published sequence. All the evaluated strains showed the presence of the LRR gene.

***Conclusion:*** These results demonstrate a possible role of this gene in the virulence of *T. vaginalis* and in the parasite infection with *Trichomonas* virus as a possible virus receptor. Further analysis of this gene and encoded protein will allow determining the role that they play in the isolates virus susceptible or resistant phenotypes.

## Introduction

*Trichomonas vaginalis* is a parasitic protozoan that is the cause of trichomoniasis, a sexually transmitted disease of worldwide importance. Annual incidence of trichomoniasis is more than 276.4 million cases worldwide (1). Both sexes can be affected by the disease. It can demonstrate a wide clinical forms variation in symptoms, from an asymptomatic presentation to severe sequelae. For women, whom are primarily affected in the vulva, vagina, and uterine cervix, and secondarily, in the urinary tract, trichomoniasis can have major health consequences. In addition, infection with *T. vaginalis* in pregnant women has been associated with premature rupture of membranes and premature delivery (2). Trichomoniasis has also been associated with an increased risk of human immunodeficiency virus (HIV) acquisition and transmission and cervical cancer (3-5). 

The host-parasite relationship is very complex, and the broad range of clinical symptoms are unlikely be attributable to a single pathogenic mechanism. The exact mechanisms of pathogenesis resulting in the wide variation in clinical presentation (from asymptomatic to severe symptomatic) has not been clearly elucidated to date (6- 7). 

In population biology, both direct and non-direct techniques are used for the identification of the genetic source of variation and the genetic locus of disease or quality strain. The non-directed approaches used random genome scanning methods initially to generated polymorphic genetic map markers, which then can be linked to traits of interest. The conversion of mapped random markers into sequence-characterized loci necessitates the isolation of the marker DNA fragment and the determination of its DNA sequence (8). 

RAPD markers are a non-directed approach in which polymorphic PCR products are amplified from genomic templates using monomer oligonucleotide primers, which generally anneals with multiple sites in different regions of the genome and amplifies several genetic loci simultaneously. Polymorphism of amplified fragment are caused by: 1) base substitution or deletion in the priming site, 2) insertion that render priming site too distant to support amplification or 3) insertion or deletion that change the size of the amplified fragment (9). In addition, RAPD markers have been demonstrated as useful genetic marker for a variety of eukaryotic organism (10). It is desirable to develop RAPD methodology because of its versatility, as the ready detection of informative loci in all species underpins the universality of the technology. 

The genetic polymorphism using the RAPD technique has been correlated with different isolates biological properties such as metronidazol susceptibility (11- 15), the presence of *Mycoplasma *in strains of *T. vaginalis* (13, 15), the presence of *T. vaginalis* virus (TVVs) infections (11-13, 16) and the clinical manifestations in patients (11, 14, 17, 18). Meade et al. (19) reported the genetic diversity in *T. vaginalis* isolates using the RFLP of cytoplasmic *hsp*70 gene and proposed that parasite isolates are organized into two clonal linages, however the authors did not correlated the phylogenetic relationships with clinical presentation. 

The association between the genetic polymorphism and clinical presentation in patients (symptomatic and asymptomatic) found by different authors (11, 14, 17, 18) demonstrate the role of the parasite in the virulence. 

In a previous paper, our group found a specific RAPD marker of 490 bp in all symptomatic isolates but not in the asymptomatic ones using the primer Tv-5 (14). When using another group of primers (OPA-1 to OPA-20) we find other two specific RAPD markers of 720 bp and 460 bp using the primers OPA-6 and OPA-11 (18). This was the first description of possible genetic markers of the virulence of the infection by *T. vaginalis.*

The aim of this study was to characterize the specific virulence RAPD markers in order to elucidate their possible biological function. 

## Materials and Methods

***Trichomonas vaginalis*** ***isolates***

Thirty-seven fresh *T. vaginalis* isolates, collected from sexually active adolescent women consulting by sexually transmitted infections in gynecobstetrics hospitals ‘‘Ramón González Coro” and ‘‘Eusebio Hernández” in Havana City, Cuba, during 1999 to June 2000, were enrolled in the study. 

Ethical approval of the study had been obtained from the Institute of Tropical Medicine Pedro Kouri and Gynecobstetric Hospitals Ethics Committee. Patients were informed of the about the study and following written informed consents from them or their parents, and clinical data were collected. 

Various laboratory tests were carried out, to determine if other sexually transmitted microorganisms, such as yeast infections, bacterial vaginosis, gonorrhoea, *Chlamydia trachomatis*, *Mycoplasma hominis*, human papilomavirus and HIV were present in patients (18, 20). The studied isolates are free of these other concomitant microorganisms. All these strains are deposited in the culture collection of the Parasitology Department from the Institute of Tropical Medicine ‘‘Pedro Kouri”, Havana, Cuba. All *Trichomonas *isolates were maintained as axenic cultures cultivated at 37 °C in TYI-S-33 Diamond’s medium (21), supplemented with 10 % heat inactivated calf serum. In the exponential growth phase parasites (2 x 10^6^) were placed in - 70 °C freezer for the later evaluation of PCR gene detection.

**Fig. 1:**
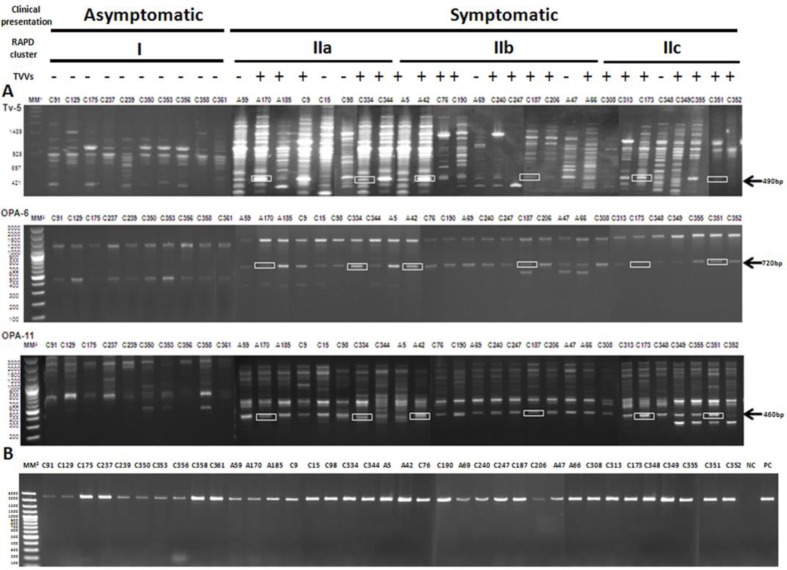
A) RAPD banding patterns obtained using primers Tv-5, OPA-6, and OPA-11. In square the RAPD marker used for sequencing. The arrows indicate the RAPD marker position. B) Agarose gel electrophoresis 2 % of PCR for the detection of the LRR family protein gene in *T. vaginalis. *The clinical presentation, RAPD cluster group and TVV infection of each isolates are shown on top. The isolate names are shown on top of each gel. MM^1^: Molecular weight marker 1 kb (Promega, USA). MM^2^: Molecular weight marker Gene Ruler™ 100 bp Plus DNA ladder (Fermentas). CP: positive control (strain ATCC 30001), NC: negative control

Medical doctors recorded symptoms (vaginal discharge, pruritus, dysuria, and dyspareunia among others) and signs (vaginal discharge, cervical, vaginal or vulvar erythema) by formal questionnaires and clinical examinations of patients. According to clinical presentation, the isolates were classified as asymptomatic (no symptoms and signs) or symptomatic (with one or more symptoms and signs). In addition, these isolates are well characterize genetically by RAPD and the infection with *Trichomonas virus* has been reported (16, 18, 22) (Fig. 1). 

***RAPD virulence markers***

Three RAPD genetic markers associated to virulence were obtained using the RAPD technique during the genetic polymorphism analysis of *T. vaginalis* isolates (14, 18). The primers Tv-5, OPA-6 and OPA-11 amplified a single, bright band of approximately 490 bp, 720 bp and 460 bp in all symptomatic isolates and was absent in the asymptomatic isolates (Fig. 1). These bands were named Tv-5_490, _OPA-6_720_ y OPA-11_460_. The putative virulent RAPD marker was successfully cloned and sequenced from six *T. vaginalis *isolates (A170, C334, A42, C187, C173, C351), choosing two isolates representative of each one of the three main RAPD genetic groups (II a, b and c) and infected with *Trichomonas *virus (Fig. 1) (16, 18, 22). 

***Cloning and sequencing RAPD virulence markers***

The RAPD-PCR fragments were excised from agarose gels with a sterile gel slicer, then extracted and purified by using QIAquick Gel Extraction Kit (Qiagen). The PCR fragments were used as a template for re-amplification with TV-5, OPA-6 and OPA-11 primers for confirmation of the right fragment. The purified PCR fragments were then directly cloned into pGEM-T Easy vector (Promega Madison) following the supplier instruction. The recombinant DNA was used to transform *E. coli *JM109 competent cells. Recombinants were identified as white colonies on LB plates with X-gal and IPTG. These colonies were further screened by taking stab of a transformant colony, boiled in 20 µl of sterile water and then 1.2 µl of the solution is used in an amplification reaction with the original primer. Plasmid DNA was isolated from putative recombinants with the QIAprep Spin Plasmid Kit (QIAgen) using the standard protocol provided by manufacturer. An aliquot of purified plasmid DNA was analyzed again by PCR, while another aliquot was digested by *Sal*I and *Nco*I restriction enzyme (Fermentas, Belgium). Sequences of the plasmid DNA were generated using the T7 and SP6 primers for sequencing both strand of the entire amplicon with dideoxy nucleotide chemistry with the ABIPRISM BigDyeTM Terminator cycle sequencing kit (PerkinElmer, Foster City, CA, USA), and analyzed on an ABI 3730 automated sequencer (PerkinElmer). The consensus sequences of each RAPD markers were obtained using the software package MEGA (23) (Molecular Evolutionary Genetic Analysis Version 4, www.megasoftware.net).

***RAPD virulence marker sequences analysis***

The sequence data were compared with other nucleotide sequences available throughout the National Center for Biotechnology Information (NCBI, USA) databases using BLASTn and BLASTx programs (Basic Local Alignment Search Tool; 24) algorithm at http://www.ncbi.nlm.nih.gov/BLAST/ of the National Center for Biotechnology Information (NCBI). Also the sequences were compared with the complete genome of *T. vaginalis* (G3 strain American Type Culture Collection PARA-98, Human, 1973, New England) using the *T. vaginalis *genome database (TrichoDB, http://trichodb.org/trichodb)

Sequence alignments were considered biologically significant when presenting E-values below 10^-^^10 ^and 10^-2 ^for nucleotide and protein sequence, respectively (25). The ORF signatures were detected using the NCBI ORF finder tool.

***Nucleic acid extraction***

The *T. vaginalis* pellet from each isolate was used for the extraction of nucleic acids (22). Genomic DNA was used as template for the detection of *T. vaginalis* Leucine Rich Repeat (LRR) family protein gene using specific PCR technique. 

***PCR amplification of Leucine Rich Repeat (LRR) family protein gene in T. vaginalis isolates***

PCR primers were designed in order to amplify the LRR family protein gene of *T. vaginalis* based on a sequence of a G3 strain reported in a GenBank with the accession number XM_001298269. Two primers, named LRRTv_F (5’-GCGACTGTGCTTATCACGAAATAG-3’) and LRRTv_R (5’-GTTTAGGG-CGGAGTATGTTGC-3’), were designed to amplify a fragment of around 2235 bp using the program Primer3 (http://simge-ne.com/Primer3). The primer sequences were checked with the Oligo Properties Calculator (http://www.basis.northwester.edu/biotools/oligocalc.html) and using BLASTn to confirm the sequence specificity. 

PCR products were amplified from the 37 *T. vaginalis* isolates. The PCR was done in 25 μl total volume containing 1x Q solution; 1× CoralLoad PCR buffer including 1.5 mM MgCl_2_; 0.4 μM of each primer; 200 μM of each deoxynucleoside triphosphate; and 1U HotStar Taq Plus DNA Polymerase (Qiagen, Hilden, Germany). In a PCR was used as a template 5µl of DNA extracted of each isolate. Negative no-template controls were always included, along with a positive control consisting of 10 ng DNA from *T. vaginalis *reference strain (ATCC 30001). The amplification conditions for the PCRs was: denaturation at 95 °C for 5 min; followed by 35 cycles of denaturation at 94 °C for 40 sec, annealing at 58 °C for 1 min, extension at 72 °C for 3 min; and a final extension step of 10 min at 72 °C. Thermal cycling was performed in a PTC-150 (MJ Research, Waltham, MA, USA). Analysis on a 2% agarose gel was used to verify the amplified product size of 2235 bp.

## Results

By aligning all clone sequences, a consensus sequence was generated which actually consisted of 466 pb, 700 pb and 440 pb (Gen-Bank accession No. JQ699181, JQ699182, JQ699183). The closest similarities of RAPD virulence marker sequences are shown in Table 1.

**Table 1:** Sequences to produce a similarity using the BLASTn program for each virulence RAPD markers

**RAPD marker**	**Blastn**	**e-value**
Tv-5_490_	XM_001298269.1 *Trichomonas vaginalis* G3 Leucine Rich Repeat family protein (TVAG_288800) partial mRNA	1e-40
	XM_001707130.1 *Giardia lamblia* ATCC 50803 Leucine-rich repeat protein 1 virus receptor protein (GL50803_5795) mRNA, complete cds	6e-14
	AF310726.1 *Giardia intestinalis* WB virus receptor protein mRNA, complete cds	6e-14
	AF310725.1 *Giardia intestinalis* JH virus receptor protein mRNA, complete cds	2e-12
OPA-6_720_	*Mesorhizobium opportunistum* WSM2075, complete genome	3.3
	Partial sequence; and homeobox protein HoxC9bb (HoxC9bb)	3.3
OPA-11_460_.	*Pinnaspis uniloba* isolate D0542A cytochrome oxidase subunit I (COI) and cytochrome oxidase subunit II (COII) genes, partial cds; mitochondrial	0.001
	PREDICTED: *Acyrthosiphon pisum* UPF0402 protein CG32590-like, transcript variant 2 (LOC100162874), mRNA	0.014

The sequence of the Tv-5_490 _RAPD marker exhibited significant sequence similarity to *T. vaginalis* hypothetical G3 LRR family protein and *G. lamblia* LRR protein 1 virus receptor protein. The sequence of each one of Tv-5_490_ RAPD marker corresponding to each isolates chosen were deposit in the GenBank with the accession numbers KF318415-KF318420. However, BLAST results revealed that OPA-6_720_ and OPA-11_460_ did not have significant similarity with any known coding nucleotide sequences. The consensus sequences of these markers correspond to non-coding region of *T. vaginalis* genome. 

The NCBI ORF finder revealed that the derived aminoacid sequence of TV-5_490 _contains a 151 aminoacids ORF signature from aminoacid leucine to arginine. The BLAST analysis of ORF showed considerable similarity to *T. vaginalis* G3 LRR family protein (accession number XP_001298270.1) (80.1 bits, E-value= 1e-15), and to *G. lamblia* LRR protein 1 virus receptor protein (accession numbers EFO64871.1, AAL37119, AAL37120, XP_001707182) (43.1 – 43.9 bits, E-value= 0.004-0.009). 

A PCR was designed in order to amplify the gene to codify the LRR family protein in *T. vaginalis*. The expected PCR product was amplified in all of isolated evaluated (Fig. 1B). 

## Discussion

The leucine rich repeat family protein constitute a group of proteins included in a large BspA like proteins, one of the three mains surface proteins family (BspA like proteins, GP63-like proteins and adhesion or others) (26, 27). The BspA-like proteins (TvBspA) are the largest gene family encoding potential surface proteins and share a specific type of LRR, named TpLRR after a membrane protein from *Treponema pallidium* (28), shared with *T. forsythensis* BspA and *Treponema denticola* LrrA proteins (29). LRR is a versatile binding motif found in a variety of proteins and is involved in protein-protein interaction (30). The TvBspA proteins could represent receptors mediating endocytosis of various host proteins with by their TpLRR likely involved in binding to ligands (31). *T. vaginalis* has over 300 candidate surface proteins, which belong to ten different families with at least one inferred transmembrane domain and share one or more features with other pathogens surface proteins (32). 

The RAPD Tv-5_490_ marker also matches homology to *G. lamblia* LRR protein 1, virus receptor protein. In *G. lamblia*, one dsRNA virus has been identified, as *Giardiavirus *(33). The time course of *Giardia *virus infection in the parasite reveals that the virus enters susceptible parasite cells via endocytosis, in which the lysosomal-like peripheral vacuoles serve a translocation ports for the virus to reach the cytoplasm (34). Sepp et al. (35) proposed that the *Giardia *virus invasion is most likely mediated by a receptor-directed endocytosis in *G. lamblia*. This putative receptor(s) is expected to be present on the surface of the virus susceptible cells, responsible for the attachment and internalization of the *Giardia *virus, but missing from the resistance cells. 

*T. vaginalis* can be infected with double stranded RNA (dsRNA) viruses known as *T. vaginalis *virus (TVVs). This virus infection induces various phenotypic changes that may affect *T. vaginalis* virulence (36, 37). In previous paper, the presence of TVVs in a group of clinical isolates was detected (22). A significant association between TVVs detection and the presence of particular clinical symptoms and signs in infected patients was demonstrated. This evidence constitutes an indicative of a possible clinical significance of TVV infection in *T. vaginalis* isolates (20). More recently, we found an association between the TVV infection and cytoadhesion level of isolates (16), as another evidence of a possible role of the virus in the virulence of human trichomoniasis. The results described by Ficharova et al. (38) support the novel concept that protozoan endobiont viruses and viral dsRNA sensors of the human host represent a critical target to exacerbate the disease through signaling of immunoinflammatory responses by human epithelial cells. More recently, Parent et al. (39) provide both mechanistic and translational insights concerning the role of *Trichomonas* viruses in aggravating diseases attributable to *T. vaginalis*. All these results demonstrate the role of the virus in the virulence of the parasite. 

The analysis of polymorphism among 37 isolates by RAPD showed significant association with the presence of TVV, demonstrating the existence of concordance between the genetic relatedness and the presence of TVV in *T. vaginalis* isolates. This result could point to a predisposition of *T. vaginalis* for the viral enters and/or survival (18). In this paper, after the characterization of RAPD markers associated to the virulence, the presence of a gene that it could be involved in the entrance of the TVVs, as it happens in *G. lamblia* with *Giardia *virus is revealed (35). Today, it is not known how the virus enters to the protozoan and whether the virus was acquired from the host in which protozoan resided. 

## Conclusion

Our results demonstrate a possible role of the parasite in the infection with TVVs. All of studied isolates, susceptible or resistant to TVVs, presents the LRR family protein gene. Further studies focused in the polymorphism characterization of the gene and it coding protein are needed, in order to determine the role of this gene in the internalization of TVVs to the protozoan. In a future, the Tv-5_490 _marker will be used to converting the RAPD marker into a single locus PCR-based marker of a sequence-characterized amplified region (SCAR) with higher sensitivity and reproducibility.
